# Management of Primary Infertility: A Comprehensive Approach With Magnetic-Activated Cell Sorting in an Asthenozoospermic Patient

**DOI:** 10.7759/cureus.53148

**Published:** 2024-01-29

**Authors:** Princee Tyagi, Akash More, Namrata Chaudhary, Shivani Khemani, Prerana Dagwar

**Affiliations:** 1 Clinical Embryology, Datta Meghe Institute of Higher Education and Research, Wardha, IND

**Keywords:** sperm dna fragmentation, icsi, art, infertility, macs

## Abstract

This report presents the cases of a 34-year-old male and a 29-year-old female who visited a fertility clinic and were experiencing primary infertility for the past three years. No previous medical history was identified in the couple. After a failed attempt of intrauterine insemination, the male partner was diagnosed with asthenozoospermia. A second attempt of intracytoplasmic sperm injection (ICSI) was performed which failed again. Subsequently, magnetic-activated cell sorting (MACS) technique was used as the sperm selection technique. The male patient’s sperms were selected through MACS before ICSI. After performing MACS, successful pregnancy was achieved, which resulted in the production of blastocysts and a positive beta-human chorionic gonadotropin test. This case report highlights the prospect of successful results, despite the presence of sperm DNA fragmentation, and a comprehensive strategy for managing infertility issues. Promoting knowledge of lifestyle factors and how they affect fertility remains an essential aspect of comprehensive infertility care.

## Introduction

According to the World Health Organization (WHO), around 8% to 12% of couples worldwide struggle with infertility, which is a serious clinical issue [1]. Infertility is referred to as one year of unprotected sexual conduct during the fertile phase of the menstrual cycle [2]. As reported, 40% of infertility cases are related to men, 40% to women, and 20% to both sexes. One of the significant factors that is effective for assisted reproductive technology (ART) success is sperm, highlighting how sperm morphology impacts patients undergoing in-vitro fertilization (IVF) and their chances of getting pregnant [3]. The use of apoptotic sperms for intracytoplasmic sperm injection (ICSI) can be the main reason for an unsuccessful ICSI cycle. One process that contributes to the beginning of DNA fragmentation is apoptosis. The damaged DNA exhibits typical apoptosis, including the translocation of phosphatidylserine (PS), the activation of caspase-3, and the reduced potential of the membrane mitochondria [3]. Recently, several studies have assessed the success of magnetic-activated cell sorting (MACS) in reducing apoptotic sperm and improving sperm quality. Based on the outcomes of these studies, other studies have investigated the sperm selection technique MACS for ART [4]. Marital discord is frequent in infertile couples, particularly when they are pressured to make medical decisions. Women who are trying to conceive are more likely to experience clinical depression, with rates being comparable to those with heart disease and cancer [1]. The technique used in laboratories that are considered the gold standard for attempting to diagnose male infertility is semen analysis. It evaluates the factors of infertility such as morphology, sperm motility, liquefaction time, seminal volume, sperm concentration, and sperm [5]. ART has been widely included in the management of infertile couples. Failures in reproductive techniques are strongly associated with sperm apoptosis [6]. Based on PS, MACS is an innovative technique that separates non-apoptotic and apoptotic spermatozoa from the sample. Based on the externalization of PS residues, MACS leveraging annexin V-conjugated microbeads curtails apoptotic spermatozoa [7].

## Case presentation

Patient information

A 33-year-old male and a 28-year-old female couple visited an infertility center situated in Maharashtra, India, with primary infertility after four years of marriage. They had been trying to conceive for the past three years.

Medical/Surgical history

This was the first time the couple sought infertility treatment, and there was no medical history.

Physical examination

There were no relevant findings while performing a physical examination. The body mass index of both the male (24 kg/m^2^) and the female (22 kg/m^2^) was normal.

Investigations

The patient underwent infertility treatment to determine the cause of infertility. The semen analysis of the male partner showed asthenozoospermia. The number of motile spermatozoa was 30%, which was below the lower reference limit. According to the WHO, it should be at least 42% or above (count: 40 million, volume: 2 mL). Other parameters were normal as per the WHO guidelines. Lab findings showed a red blood cell count of 4,100,000 µ/L and hemoglobin of 13 g/dL, and human immunodeficiency virus, hepatitis B surface antigen, and hepatitis C virus were non-reactive. The female partner underwent a transvaginal ultrasound to assess other structural abnormalities, which were found to be normal. The hormonal profile showed normal anti-müllerian hormone, follicle-stimulating hormone, and luteinizing hormone levels.

Diagnosis

To diagnose the cause of infertility, the husband’s semen analysis was done. The diagnosis showed that the potential cause of infertility was asthenozoospermia. His wife was normal with all diagnostic parameters with no relevant findings of infertility. Hence, this was a case of primary infertility.

Treatment

Investigations showed that the primary cause of infertility was asthenozoospermia in the male partner. First, intrauterine insemination (IUI) was attempted using a swim-up method for sperm selection; however, it was unsuccessful. Subsequently, he followed the medications prescribed by the doctor (folic acid and multivitamins once a day) throughout the month. After a month, his semen analysis was performed, and it was found that motility was slightly elevated by 30% to 36%. After a month, the gynecologist advised ICSI as the next procedure after a failed IUI cycle. Initially, the female partner underwent controlled ovarian stimulation to retrieve the ovum. Gonadotropins were administered for ovarian stimulation, and the ovarian response was monitored by ultrasonography scans and serum estradiol levels. Further, when a desirable size of over 18 mm was achieved by the follicles, the trigger was given. Oocyte retrieval was done after 36 hours of the trigger.

Throughout the procedure of oocyte retrieval, 14 oocytes were retrieved and the ICSI procedure was performed with the female partner’s oocytes and the male partner’s sperms selected by density gradient. The needle aspirated spermatozoa directly injected into oocytes. After ICSI, the injected oocytes were placed under a suitable culture medium for blastocyst formation following different cell stages of the cell. However, again the cell growth was arrested, and spermatozoa failed to fertilize oocytes.

Later, the andrologist suspected DNA fragmentation, and a sperm test was done. Overall, 34% sperm DNA fragmentation (SDF) was diagnosed which was slightly above the reference range (<30%).

**Figure 1:**
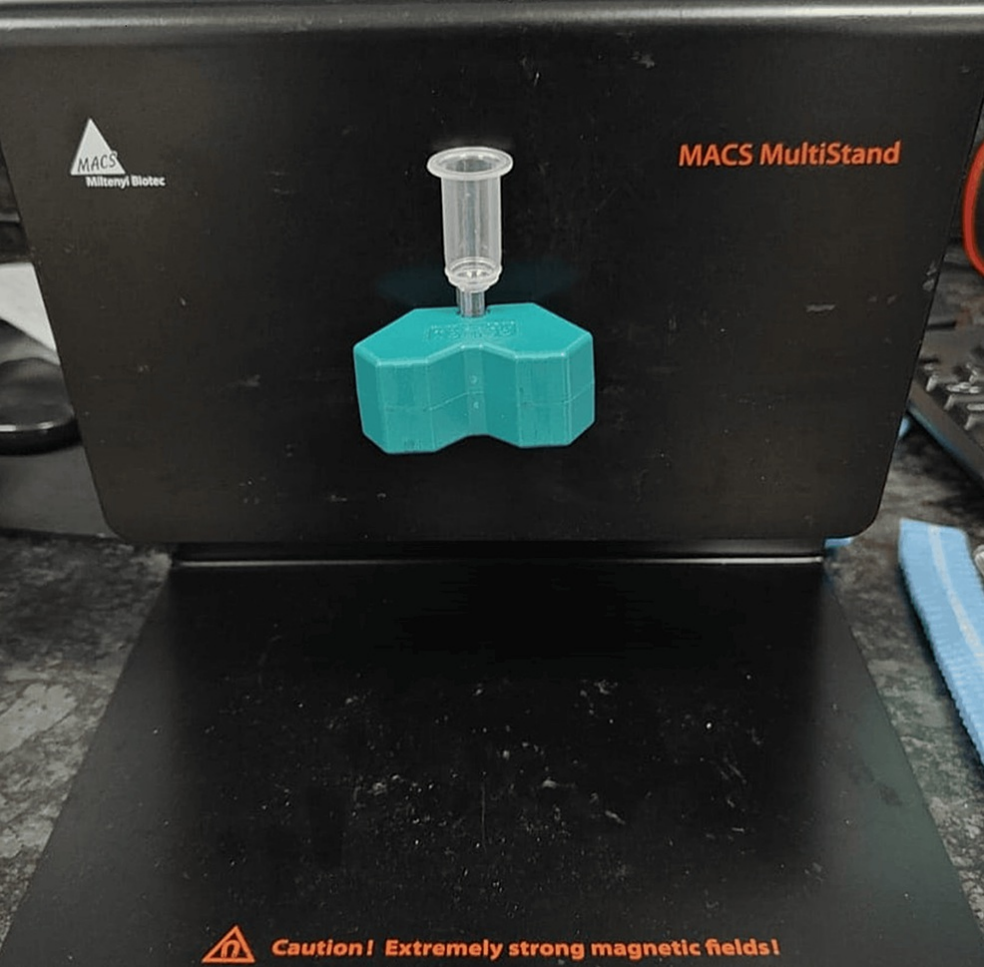
Magnetic-activated cell sorting device used for sperm selection.

We counseled the patients regarding the issue and the patient decided to opt for the next cycle. The second cycle of oocyte pick up (OPU) was continued for the next cycle as OPU was done, and this time a total of 12 oocytes were retrieved. As the DNA fragmentation index (DFI) showed that the patient’s sperm had damaged DNA, we decided to use MACS (device shown in Figure 1) as a sperm selection technique for ICSI after semen processing. The novel technique known as MACS is used to distinguish apoptotic sperms with a higher percentage of fragmented DNA. After sperm selection, ICSI was again performed using good-quality sperms cultured under a favorable environment. This time embryos progressed from the four-cell stage embryo to the eight-cell embryo stage. Eventually, fresh embryo transfer was performed on the day fifth. After 14 days of the embryo transfer, the beta-human chronic gonadotrophin (β-hCG) test was done and the results of the β-hCG test were positive. On the successful results of medical treatment, the couple was pleased and conveyed gratitude.

Follow-up

On the 14th day, the pregnancy test was again positive. For continuous follow-ups, the female was advised for regular checkups and prescribed vitamins and certain medications. Fetus development and health were regularly monitored. At the moment of writing, she is at 25 weeks of gestation with normal growth and a developing fetus.

## Discussion

According to a study done by Chauhan et al., pregnancy is possible even with DNA fragmentation of sperms. Similarly, in other underdeveloped countries, infertility or childlessness is a huge societal concern. As frequently only women are accused of not having children, recent studies demonstrate that men can be accountable for infertility [1]. In our report, the cause of infertility was the male partner.

Idiopathic infertility is the outcome of a persistent decline in male fertility with age that cannot be due to an exact cause. Male infertility can be caused by several factors that can influence the SDF, including genetic disorders, oxidative stress, varicocele, systemic illnesses and infections, and modified lifestyle. After reaching the ages of 35-40 years, there is a reduction in the quality of the semen, and an elevation in the SDF is observed. Routine semen analysis gives an enormous amount of information on sperm parameters [8]. The diagnosis of infertility in males begins with performing conventional semen analysis which gives an overview of the overall wellness of the male reproductive organs. The WHO guideline is frequently used to determine semen volume, motility, pH, vitality, sperm concentration, and morphology [9]. We also conducted a semen analysis and found that the patient was asthenozoospermic. Simultaneously, SDF was also performed, and the DFI was higher than the lower reference value.

Research from around the world has revealed that people have a lack of awareness regarding the biological aspects of conception and the most fertile phase of the menstrual cycle. The effects of alcohol consumption, smoking, work stress, and other aspects of life on the potential for conception are not well understood in both sexes [10]. The growing significance of male infertility and SDF in management and diagnosis lies in the fact SDF may have a major effect on male fertility and reproductive outcomes [11]. In our case, the cause of infertility was likely due to higher DFI.

During sperm preparation, MACS is a procedure that separates spermatozoa with externalized PS utilizing annexin V-conjugated microbead technology [12]. The sensitivity of the sperm to DNA fragmentation can also demonstrate the lack of a DNA repair mechanism. Studies have demonstrated that compared to testicular sperm, epididymal and ejaculated sperm have more DNA fragmentation [13]. According to the systematic review and meta-analysis by Gil et al., using MACS as a sperm selection technique increases the success rates of ART for couples receiving it. MACS is a safe and effective way to select viable sperm that consistently yields positive results. When combined with other accepted sperm selection techniques in ART, this strategy may increase the possibility of conception [4]. In a similar vein, using MACS for sperm selection, led to positive results in this case and the patient conceived.

## Conclusions

Our case report demonstrated a couple who experienced primary infertility, mainly attributed to asthenozoospermia. Lifestyle factors, alcohol intake, and increased SDF in the male partner might have additionally contributed to the infertility. The male patient underwent the MACS technique which contributed to blastocyst formation and the selection of spermatozoa used for ICSI. Continuing with standard procedures, incorporating MACS turned into an essential method for managing SDF throughout ART, particularly in IVF. However, this is a single study and large-scale studies are needed for accurate results.
